# Complete Preoperative Evaluation of Pulmonary Atresia with Ventricular Septal Defect with Multi-Detector Computed Tomography

**DOI:** 10.1371/journal.pone.0146380

**Published:** 2016-01-07

**Authors:** Jingzhe Liu, Hongyin Li, Zhibo Liu, Qingyu Wu, Yufeng Xu

**Affiliations:** 1 Department of Radiology, First Hospital of Tsinghua University, Beijing, China; 2 The Heart Center, First Hospital of Tsinghua University, Beijing, China; 3 Department of Radiology, Peking University First Hospital, Beijing, China; University of Louisville, UNITED STATES

## Abstract

**Objective:**

To compare multi-detector computed tomography (MDCT) with cardiac catheterization and transthoracic echocardiography (TTE) in comprehensive evaluation of the global cardiovascular anatomy in patients with pulmonary atresia with ventricular septal defect (PA-VSD).

**Methods:**

The clinical and imaging data of 116 patients with PA-VSD confirmed by surgery were reviewed. Using findings at surgery as the reference standard, data from MDCT, TTE and catheterization were reviewed for assessment of native pulmonary vasculature and intracardiac defects.

**Results:**

MDCT was more accurate than catheterization and TTE in identification of native pulmonary arteries. MDCT is also the most accurate test for delineation of the major aortopulmonary collateral arteries. The inter-modality agreement for evaluation of overriding aorta and VSD were both excellent. In the subgroup with surgical correlation, excellent agreement was found between TTE and surgery, and substantial agreement was also found at MDCT.

**Conclusion:**

MDCT can correctly delineate the native pulmonary vasculatures and intracardiac defects and may be a reliable method for noninvasive assessment of global cardiovascular abnormalities in patients with PA-VSD.

## Introduction

Pulmonary atresia with ventricular septal defect (PA-VSD) is defined as a group of cardiac malformation characterized by lack of luminal continuity, and absence of blood flow between right ventricle and pulmonary artery, with egress of blood from right ventricle (RV) occurring through a ventricular septal defect [1–3]. Owing to atresia of the pulmonary valve, the pulmonary vascular bed may be supplied rom several sources such as aortopulmonary collateral arteries (APCAs) and patent ductus arteriosus (PDA). Complete delineation of the global cardiovascular anatomy (including native pulmonary artery, APCAs, PDA, and intracardiac malformation) is therefore essential to patient management [4–5].

The main preoperative goals of imaging are as follows: (a) visualization of the native pulmonary arteries; (b) assessment of the numbers, origin and course of the APCAs; and (c) characterization of the intracardiac malformation. Traditionally, the first two goals are typically addressed at cardiac catheterization with conventional cineangiography, and the latter is assessed at echocardiography. Although echocardiography is regarded as an initial screening diagnostic modality in patients with PA-VSD and provides accurate information about the intracardiac malformations, it is often of limited value in delineation of the native pulmonary artery and APCAs because of poor acoustic windows [6–8]. Sole echocardiographic evaluation might not provide adequately comprehensive anatomic demonstration for surgical planning. Cardiac catheterization is an invasive procedure involving radiation exposure and injection of iodinated contrast agent and can be associated with a small but definable complication [9]. So a noninvasive alternative imaging modality, which can fulfill three preoperative goals, would be advantageous for serial assessment and reduced risk and cost. Current fast MR imaging and MR angiographic acquisition techniques with bolus injections of contrast material can provide images of the pulmonary vasculature that are similar to those obtained at multi-detector computed tomography (MDCT), but clinical application of MRI is limited because of the following disadvantages [10–13]. Due to long scan time and discomfort, MRI may be difficult for cyanotic patients or those with cardiorespiratory compromise. Pediatric patients often require deeper sedation during MRI scan, which may be associated with more risks. MRI cannot be used in many postoperative patients with pacemakers, surgical clips, vascular grafts and stents and the critically ill patients in the intensive care unit. Another limitation of the MRI is that the more peripheral branches of the pulmonary arteries are not delineated because of limited spatial resolution. In addition, turbulence in stenotic blood vessels and anastomoses leads to underestimation of their caliber at MRA. Most surgeons in our medical center, therefore, currently favor MDCT over MRI for the evaluation of patients with PA-VSD, especially the unstable postoperative patients. Previous studies with small sample size have revealed that MDCT is equivalent or superior to cardiac catheterization for the preoperative and postoperative characterization of pulmonary vasculature [14–15]. The clinical utilization of MDCT was limited for the assessment of intracardiac anatomy in most previous papers [16]. However, with the development of MDCT and increased experiences, the evaluation of intracardiac anatomy has been further improved. The present study, therefore, was retrospectively undertaken to compare MDCT with cardiac catheterization and echocardiography in preoperative evaluation of the global cardiovascular anatomy in patients with PA-VSD.

## Materials and Methods

### Patient Population

**Fig 1:**
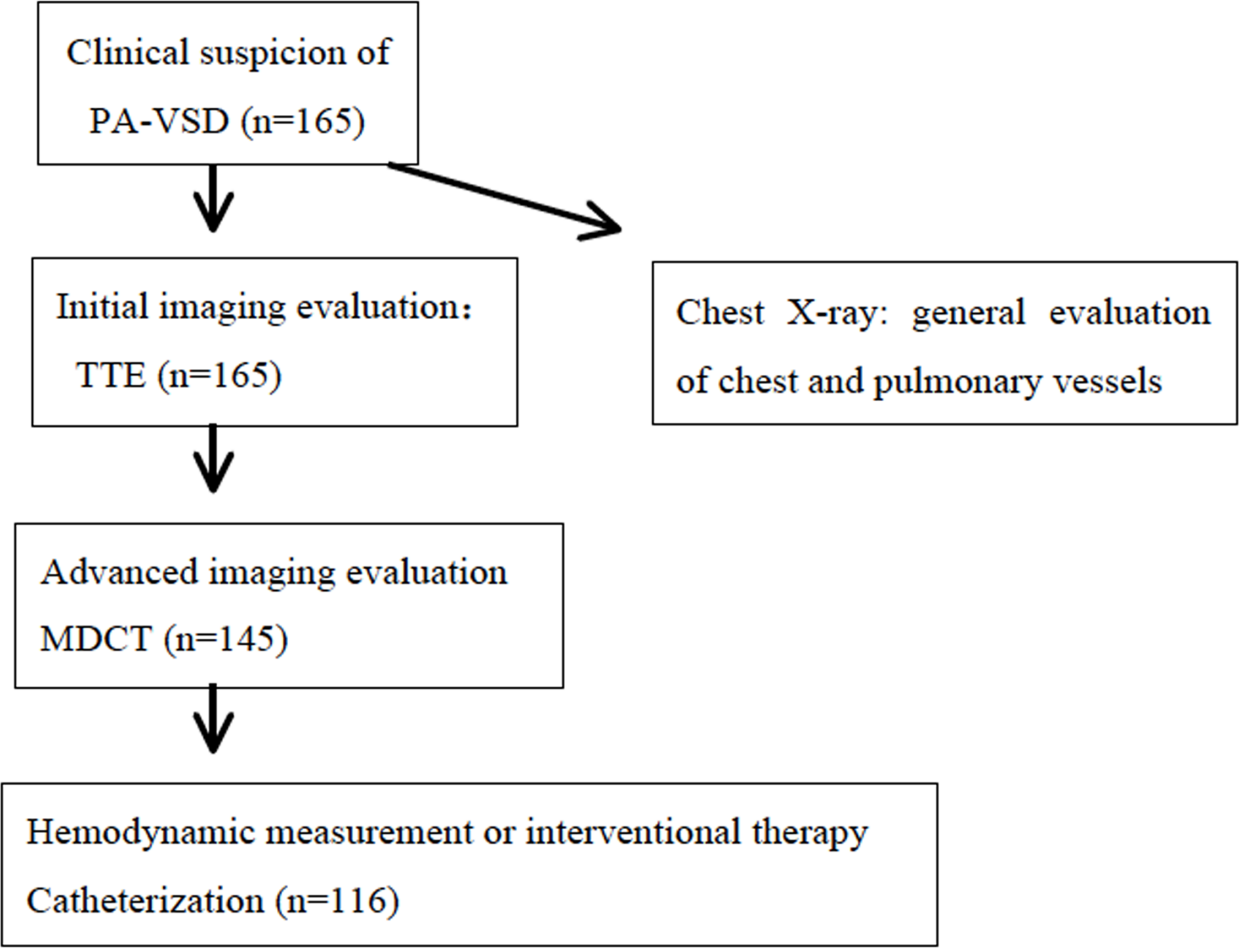
The diagnostic algorithm used for patients with PA-VSD.

A total of 165 consecutive patients with clinical suspicion of PA-VSD between July 2013 and December 2014 at First Hospital of Tsinghua University were retrospectively reviewed. Patients who fulfilled the following criteria were included in this study: 1) underwent all three imaging examination including transthoracic echocardiography (TTE), MDCT and cardiac catheterization prior surgery. 2) Interval time was less than 1 month and without an interim surgical procedure between imaging examinations. 3) Diagnosis of pulmonary atresia with ventricular septal defect (PA-VSD) confirmed by surgery. The diagnostic algorithm used for patients with PA-VSD in our hospital is outlined in Fig 1. All patients with clinical suspicion of PA-VSD initially start with echocardiography. For patients with pulmonary artery or APCAs difficult to display by echocardiography, MDCT is the preferred next step in imaging evaluation. Catheterization may be performed when hemodynamic measurement or interventional therapy is needed. In addition chest X-ray, which is not included in this study, is also a common initial imaging examination to provide general information about lung and pulmonary vessels. As a result, 116 patients were enrolled in this study. They ranged in age from 2 months to 16 years (median age, 3 years and 4 months) and in weight from 3.4 to 48 kg (median weight, 11.4kg). Fifty-two(44.8%) were boys, and 64(55.2%) were girls.

All MDCT scans were clinically indicated and written informed consent was obtained from all patients or their legal guardians. The institution ethics committee of First Hospital of Tsinghua University approved review of the medical records and computer databases.

### Patient preparation

Before MDCT examination, no food was given orally for 4–6 hours in all patients. Flexible venous cannula catheters were placed in the antecubital vein or the peripheral vein of the foot. Patients younger than 5 years old were sedated by the pediatric anesthesia service using oral chloral hydrate.

### MDCT imaging protocol

All patients underwent imaging with a 64-slice CT scanner (Brilliance 64, Philips, Eindhoven, Netherlands) using the following parameters: detector collimation 32×0.6 mm, slice acquisition 64×0.6 mm by means of z-flying focal spot, pitch 1.173:1, tube voltage 80–120 kV, and tube-current-time product 55–300 mAs. An IV iopromide (Ultravist 370; Schering, Berlin, Germany) (2.0 mL/kg, 80 mL maximum volume) was injected by mechanical power injector (Injektron CT2, Medtron, France) followed by a 10-30ml saline flush at rates ranging from 1.5 to 2.5 ml/s. Scanning began 14–16 sec after beginning injection of contrast material in the peripheral vein of the foot and 10–11 sec after injection in the antecubital vein, which was empirically determined on the basis of estimated times for circulation from the injection site to the aorta.

Images in all infants and young children were acquired during quiet respiration, but in cooperative older children or adults, image acquisitions were performed during a single breath hold.

MDCT data were reviewed using a combination of MPR, MIP, and 3D VR, which were performed on workstation. All CT images were reviewed by two attending cardiac radiologists in consensus who had no knowledge of the surgical findings at the time of review.

On all CT images, we visually rated the presence of the following: (1) main pulmonary artery; (2) pulmonary artery confluence; (3) right and left branch pulmonary artery (4) branch pulmonary artery stenosis; (5) stenosis of the origin of aortopulmonary collateral vessels. Stenosis was defined as a focal reduction of more than 50% of the luminal diameter. In addition, the numbers and courses of all visualized APCs were recorded. (6) patent ductus arteriosus.

The presence of overriding aorta was rated and the degree of "override” was noted as: ≤50%, 50%~90% and >90%. The morphology of ventricular septum defect (VSD) was recorded as one of three types: perimembranous, muscular and subarterial VSD.

### Cardiac Catheterization

Angiographic technique was tailored to the clinical indication for the examination. Therefore, it varied considerably throughout the study. It generally included aortography in the descending aorta, ascending aorta, or aortic arch, with selective injections of contrast agent into the collateral vessels and pulmonary arteries. One investigator reviewed the angiograms and recorded the same anatomical variables as described above for MDCT.

### Transthoracic Echocardiography (TTE)

Transthoracic echocardiography was obtained in all patients. The examination protocol included two-dimensional and Doppler imaging from the subxiphoid, apical, parasternal, and suprasternal views. Studies were recorded on 1.27 cm (0.5 in.) super-VHS videocassette tapes. An investigator who was unaware of the findings at surgery reviewed all preoperative echocardiography, with particular attention to the morphology of interventrial septum, the pulmonary artery, the APCAs and PDA.

### Surgery

Surgical procedures were undertaken in all patients, including complete repair in 23 patients (19.8%), Glenn shunt in 6 patients (5.2%), Blalock-Taussig shunt in 87 patients (75%). The surgical reports in all patients were retrieved and the findings presented in these were used as the reference standard.

### Statistical analysis

To assess the accuracy of MDCT, TTE and cardiac catheterization for displaying the pulmonary vasculatures, three image modalities findings regarding the anatomic variables detailed above were recorded and analyzed for discrepancies, using findings at surgery as the reference standard. True-positive, false-positive, true-negative, and false-negative scores for each visualization criterion were calculated. By using these data, test parameters (sensitivity, specificity, positive and negative predictive values, and accuracy) were calculated for each visualization criterion. As well, all visualization scores for native pulmonary artery were summed and combined to generate overall summary test parameters.

By using Kappa test, the inter-modality agreement for detection of intracardiac abnormality was analyzed. In the subgroup of patients who underwent complete repair of PA-VSD, the agreement between each imaging modality and surgical results was also estimated. The κ values were interpreted as follows: 0.20 or less indicated poor agreement; 0.21–0.40, fair agreement; 0.41–0.60, moderate agreement; 0.61–0.80, substantial agreement; and 0.81–1.00, excellent agreement.

## Results

Apart from occasional nausea and vomiting after the injection of contrast material (which resolved spontaneously) in 3 patients (2.6%), there were no marked complications associated with the MDCT examinations. Angiographic complications were not specially rated during this study.

### Pulmonary Artery

**Fig 2:**
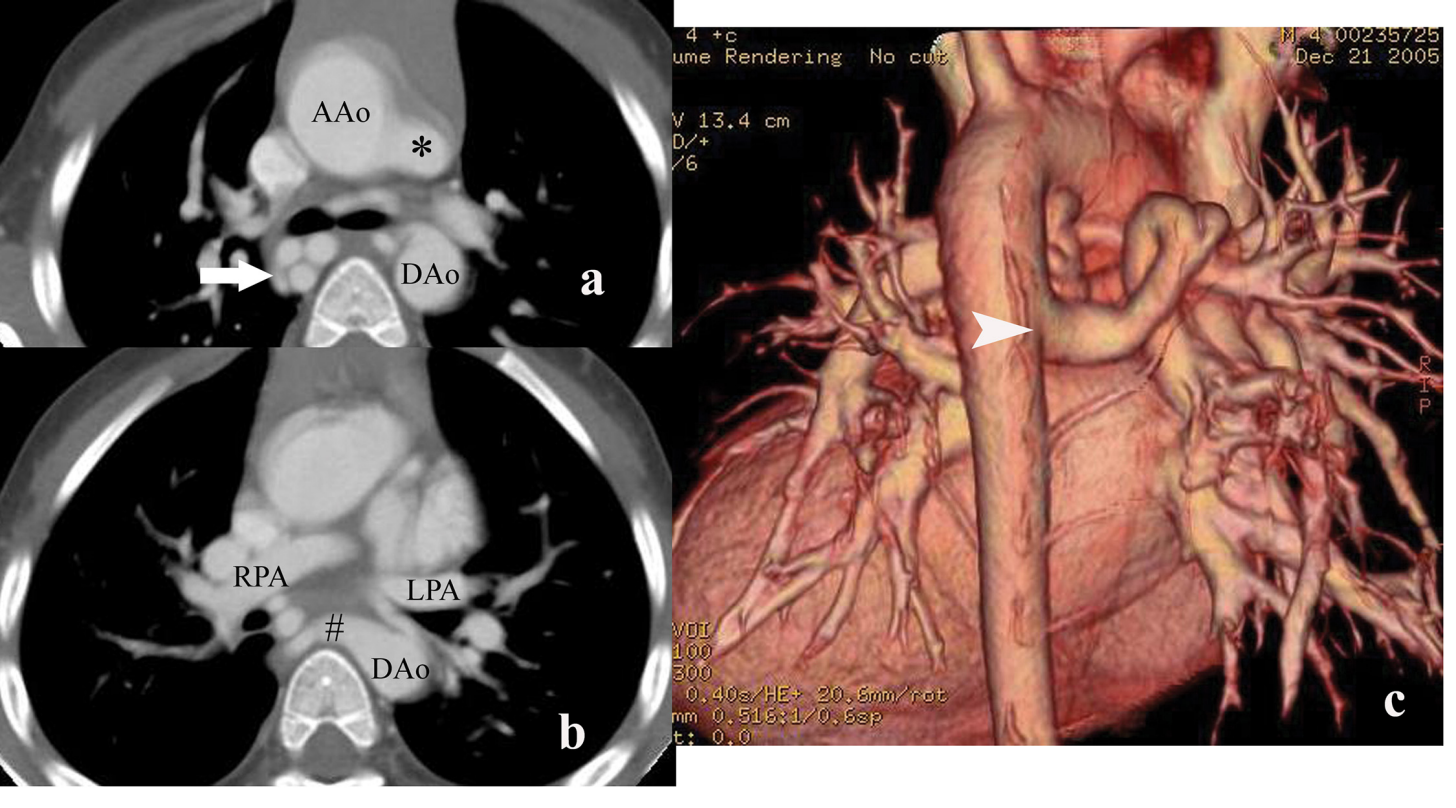
MDCT in a 4-year-old boy with PA-VSD. (a) Axial CT shows the patients has patent ductus arteriosus(*). Some MAPCAs (white arrow) from the descending aorta (DAo) to the right lung. (b) Axial CT demonstrates good-sized right pulmonary artery (RPA) and left pulmonary artery(LPA). The orifice of a MAPCA (#) is depicted clearly. (c) 3D Volume-rendered image (posterior view) shows a MAPCA (white arrowhead) from the descending aorta to the right lung.

Findings at surgery indicated that both branch pulmonary arteries were absent in 34 patients (29.3%). In 4 patients (3.4%), only left pulmonary artery was identified in the hilum at surgery, and in 2 patients (1.7%) only right pulmonary artery was identified. Both branch pulmonary arteries were present in 76 patients (65.5%) (Fig 2), but they were not confluent in 8 patients (8/76). In the remaining 68 patients (68/76), the branch pulmonary arteries had confluence. The main pulmonary arteries were absent in 83 patients (71.6%). Stenosis was present in 21 branch pulmonary arteries in 18 patients. These stenoses were located in the proximal left branch pulmonary artery in 10 patients, in the right pulmonary artery in 5 patients and bilateral in 3 patients.

The results of our comparative analysis of MDCT, TTE and cardiac catheterization versus surgery are summarized in the Table 1. The presences of pulmonary arteries were correctly identified on MDCT in all patients. And all stenosis were well depicted at MDCT, whereas one additional stenosis in the right branch pulmonary artery at MDCT was not confirmed at surgery. There were 24 discrepancies (22 False native, 2 False positive) between cardiac catheterization and surgery, and 91 (75 False native, 16 False positive) for TTE. For overall pulmonary artery characterization, MDCT was slightly more accurate than catheterization (99.8%versus 95.9%). The results showed that TTE was the least accurate modality. The low accuracy of echocardiography (85.9%) is primarily attributable to a low sensitivity (72.9%).

**Table 1:** Findings at MD CT, TTE, and Cardiac Catheterization Compared with Findings at surgery.

Parameter		No. of Surgical Findings				Test Parameters				
	Technique	True- Positive	False- Negative	True- Negative	False- Positive	Sensitivity	Specificity	PPV	NPV	Accuracy
Main PA	MDCT	33	0	83	0	100%	100%	100%	100%	100%
	Catheterization	30	3	82	1	90.9%	98.8%	96.8%	96.5%	96.6%
	TTE	26	7	80	3	78.8%	96.4%	89.7%	92.0%	91.4%
Right/ Left PA	MDCT	158	0	74	0	100%	100%	100%	100%	100%
	Catheterization	146	12	74	0	92.4%	100%	100%	86.0%	94.8%
	TTE	112	46	70	4	70.9%	94.6%	96.6%	60.3%	78.4%
PA confluence	MDCT	68	0	48	0	100%	100%	100%	100%	100%
	Catheterization	64	4	48	0	94.1%	100%	100%	92.3%	96.6%
	TTE	53	15	43	5	77.9%	89.6%	91.4%	74.1%	82.8%
PA stenosis	MDCT	18	0	97	1	100%	99.0%	94.7%	100%	99.1%
	Catheterization	15	3	97	1	83.3%	99.0%	100%	97.0%	96.6%
	TTE	11	7	94	4	61.1%	95.9%	73.3%	84.7%	90.5%
Overall native PA	MDCT	277	0	302	1	100%	99.7%	99.6%	100%	99.8%
	Catheterization	255	22	301	2	92.1%	99.3%	99.2%	93.2%	95.9%
	TTE	202	75	287	16	72.9%	94.7%	92.7%	79.3%	85.9%
MAPCA stenosis	MDCT	96	2	225	3	98.0%	98.7%	97.0%	99.1%	98.5%
	Catheterization	85	13	222	6	86.7%	97.4%	93.4%	94.4%	94.2%
	TTE	NA	NA	NA	NA	NA	NA	NA	NA	NA
PDA	MDCT	47	0	69	0	100%	100%	100%	100%	100%
	Catheterization	47	0	69	0	100%	100%	100%	100%	100%
	TTE	47	0	69	0	100%	100%	100%	100%	100%

PA = pulmonary artery; PPV = postive predictive value; NPV = negative predictive value; TTE = transthoracic tchocardiography; MAPCA = major aortopulmonary collateral artery; PDA = patent ductus arteriosus; NA = not assess.

### APCAs and PDA

**Fig 3:**
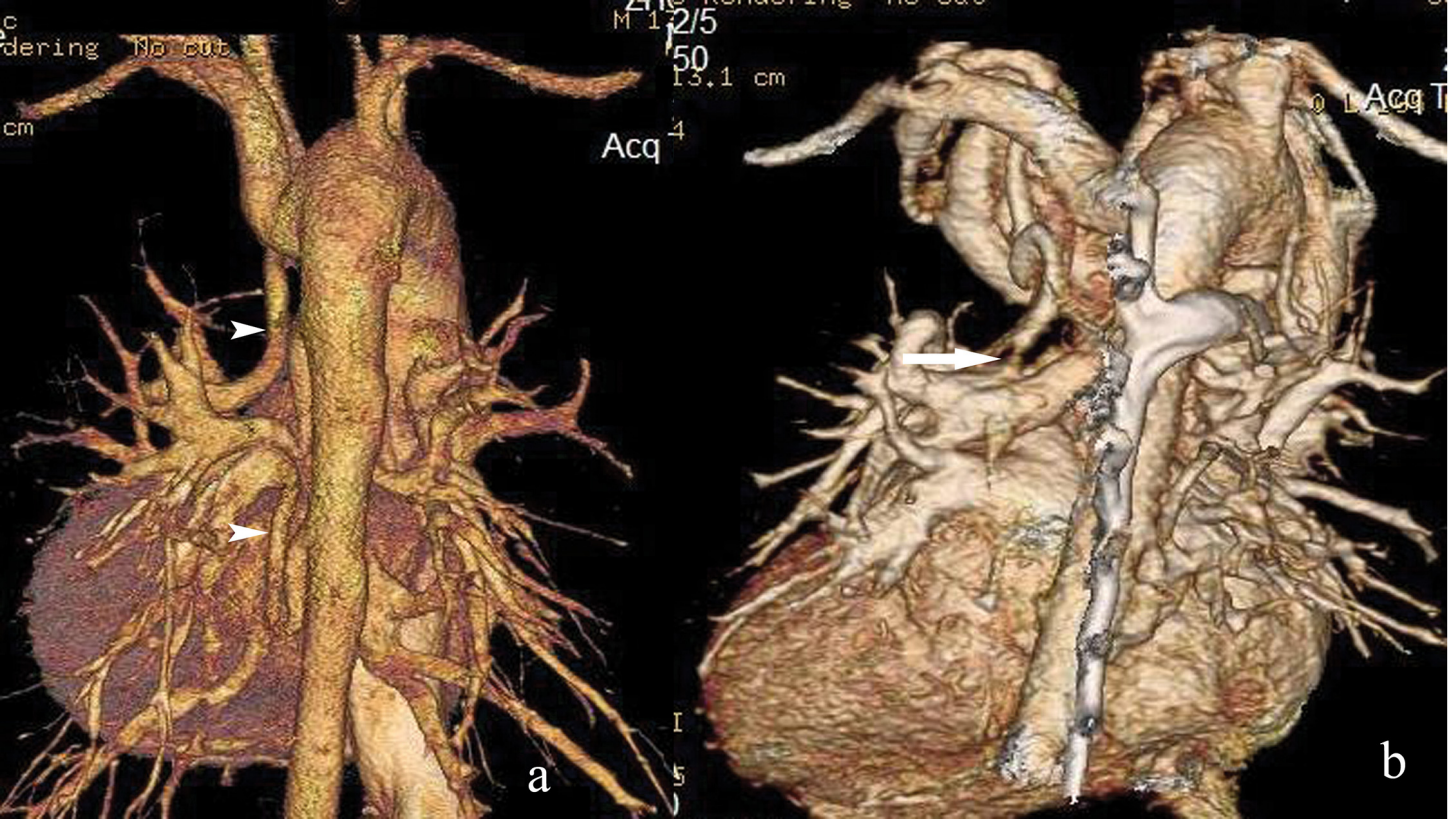
(a) 3D Volume-rendered image (posterior view) shows MAPCA (white arrowhead) from descending aorta and left subclavian artery in a 2-year-old boy with PA-VSD. (b) Another 6-year-old girl with PA-VSD. 3D Volume-rendered image (posterior view) demonstrates a stenosis (white arrow) on a MAPCA from left subclavian artery.

At surgery, a total of 326 major aortopulmonary collateral arteries (APCAs) were identified in 105 patients (90.5%) and 11 patients (9.5%) had no MAPCAs (Fig 3). Of these 326 MAPCAs that were deemed surgically important, 135 (41.4%)supplied the left lung, 147 (45.1%)supplied the right lung, and 44 (13.5%)supplied both lungs. At MDCT, all 326 surgically important aortopulmonary collateral vessels were correctly identified. Catheterization did not delineate 15 MAPCAs (7 from abdominal aorta, 4 from subclavian artery and 4 from innominate artery) in 12 patients (accuracy, 95.4%). Ninty-seven patients (97/105, 92.3%) with MAPCAs were identified at TTE, however, the numbers and courses of which could not be displayed clearly.

Significant stenoses of the MAPCAs were present in 98 MAPCAs in 34 patients at surgery. The stenosis in the MAPCAs was well depicted by MDCT (sensitivity, 98.0%; specificity, 98.7%), which is more sensitive than catheterization (sensitivity, 86.7%; specificity, 97.4%). Because TTE could not delineate the number and course of the MAPCAs, the stenosis of MAPCAs was not rated for echocardiograms. Forty-seven patients were confirmed to have PDA at surgery. The accuracies of MDCT, catheterization and TTE for detection of PDA were all 100%.

### Intracardiac Abnormality

**Fig 4:**
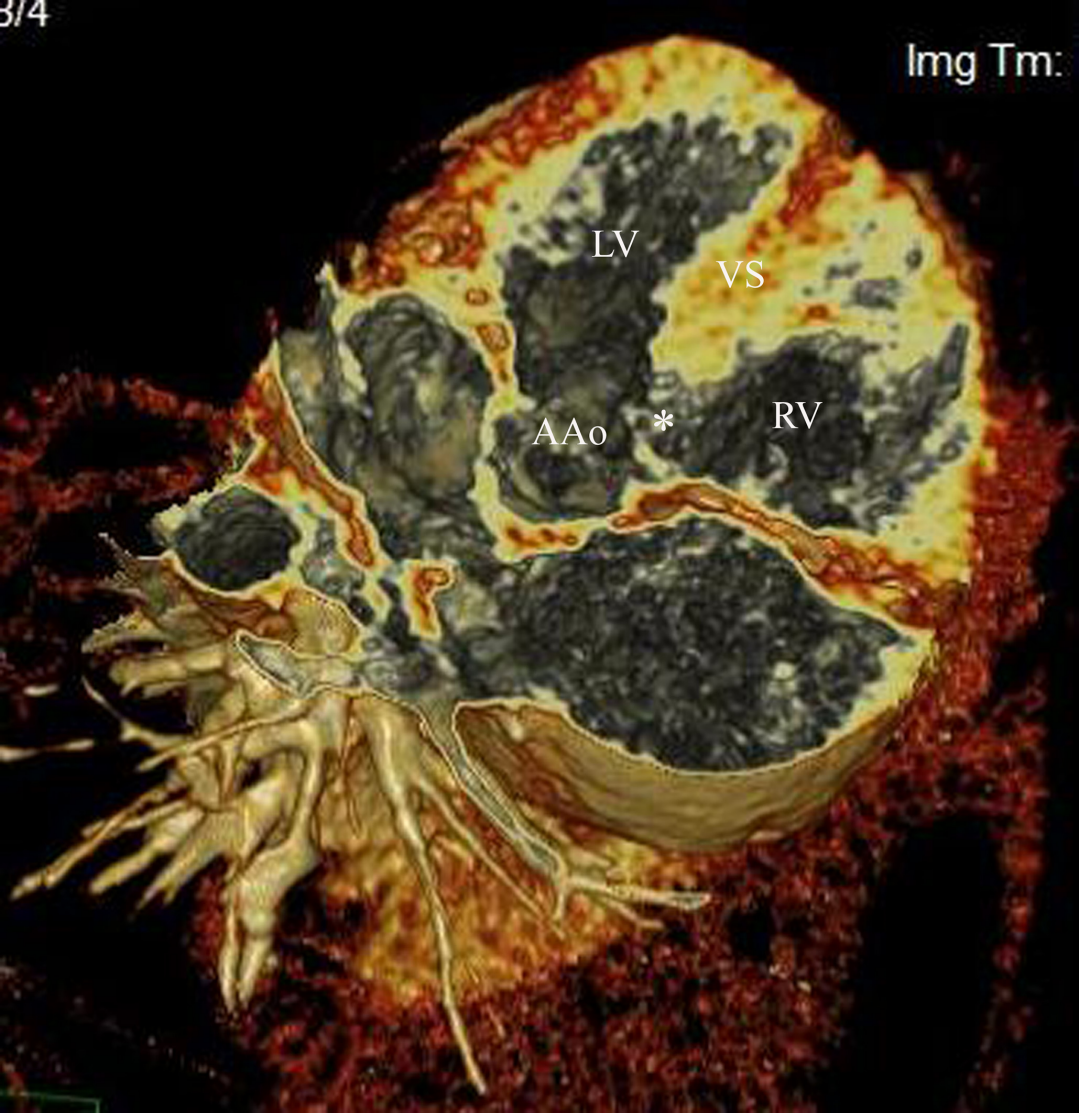
Imaging in a 3-year-old boy with PA-VSD. 3D Volume-rendered image with electronic dissection, which remove contrast from the cardiac chambers, shows the defect (*) in ventricle septum (VS) and the overriding aorta (<50%). AAo indicates ascending aorta.

Table 2 and Table 3 summarize the results and the agreement analysis of the TTE, MDCT and catheterization for characterization of intracardiac defects. The MDCT showed the degree of overriding is no more than 50% in 77 patients, more than 90% in 15 patients (Fig 4). As illuminated in Table 3, there was excellent agreement (κ = 0.824) between MDCT and TTE in the delineation of overriding aorta, and excellent agreement (κ = 0.803) between MDCT and catheterization. At MDCT, the types of ventricular septal defect (VSD) were as follows: perimembranous in 84 cases; muscular in 14 cases; and subarterial in18 cases. Excellent agreement (κ = 0.881) was found between MDCT and TTE for classification of VSD, and excellent agreement (κ = 0.851) between MDCT and Catheterization.

**Table 2:** Results of three imaging madalities for intracardiac abnormalities.

	Overridding degree			VSD		
	≤50%	50%~90%	>90%	perimembranous	muscular	subarterial
MDCT	77	24	15	84	14	18
Catheterization	72	29	15	88	16	12
TTE	75	25	16	83	15	18

VSD = ventricular septal defect; TTE = transthoracic echocardiography

**Table 3:** Inter-modality agreements for intracardiac abnormalities.

Inter-modality	Overridding degree	VSD
	κ value	κ value
MDCT vs. TTE	0.824	0.881
MDCT vs. Catheterization	0.803	0.851

VSD = ventricular septal defect; TTE = transthoracic echocardiography

### Subgroup of patients with surgical correlation

Twenty-three patients underwent complete repair of PA-VSD. For displaying the degree of aorta overridding, excellent agreement (κ = 0.824) was found between TTE and surgery, substantial agreement (κ = 0.723) was found at MDCT and catheterization (κ = 0.613). Agreements for classification of VSD at TTE, MDCT and catheterization were excellent (κ = 0.835), substantial (κ = 0.746), and substantial (κ = 0.624) respectively.

### Additional Findings at MDCT

Additional findings on MDCT included coronary artery abnormalities (6 cases), coronary artery to pulmonary artery fistula (2 cases), coronary artery to right ventricle fistula (1 case), inferior vena cava abnormalities (4 cases) and pulmonary venous abnormalities (3 cases). These findings were not prospectively reported at angiography. In addition, other system diseases were also detected at MDCT, which included hiatal hernia (2 cases), pulmonary sequestration (1 case), pneumonia (14 cases), pulmonary tuberculosis (3 cases), adrenal pheochromocytoma (1 case), spina bifida (2 cases).

## Discussions

The results of this study showed that MDCT is a reliable exploratory test for noninvasive assessment of global cardiovascular anatomy in patients with PA-VSD, in comparison with transthoracic echocardiography and catheterization.

Findings from the current study indicate that for the evaluation of branch pulmonary artery, the accuracy of MDCT surpassed that of catheterization. Additionally MDCT showed more collateral vessels than catheterization. MDCT also is the most accurate test for the diagnosis of stenosis in the pulmonary artery and aortopulmonary collateral artery. The advantages of MDCT include large imaging area and rapid data acquisition in a single breath-hold, which allow a full delineation of the entire pulmonary vasculature and aortopulmonary collateral arteries. At MDCT, all cardiac chambers and pulmonary vessels are opacified with one injection of contrast material. Difficult-to-catheterize vessels, such as hypoplastic branch pulmonary arteries, which are considered as “absent” at angiography, can be depicted at MDCT [17]. Furthermore MDCT can offer high spatial resolution and cross-sectional images with 3D reconstruction (as depicted in Figs 2 and 3), which has proved helpful in the evaluation of complex overlapping vascular structure that would superimpose on projection radiographs [18].

In the present study, the results showed TTE was limited for overall pulmonary artery characterization. The low accuracy of echocardiography is primarily attributable to low sensitivity, resulting from a relative large number of cases whose branch pulmonary arteries could not be satisfactorily visualized. Restricted acoustic windows and operator dependency limited the reliability of TTE for visualization of the branch pulmonary arteries. And the interposition of aerated lung tissue precludes the depiction of most aortopulmonary collateral vessels [19,20]. TTE is considered the initial and most suitable image modality to depict intracardiac defects. In this study, TTE remains the most accurate test for delineation of intracardiac abnormality. The new MDCT machine produces a volume of dataset with an isotropic spatial resolution less than 0.5 mm for each voxel. Given this high spatial resolution, MDCT offers volume-rendering images of the entire heart. Moreover, new reconstruction technique, which can carry out an “electronic dissection” of the heart in any possible plane and remove obstructive structures and contrast from the image, may display the intracardiac anatomy such as ventricular or atrial septal defects distinctly. This technique is a reasonable tool for imaging anatomic features of complex congenital heart disease.

In our study, MDCT frequently showed additional findings, such as coronary abnormalities, pulmonary or systemic venous abnormalities, pleural fluid collections, pulmonary parenchymal disease, and airway compression, those were important in the perioperative assessment and that were not routinely or well depicted at angiography and TTE.

Although MDCT can provide limited functional cardiac assessment (such as ejection fraction, cardiac index, and myocardial mass), it remains a relatively static imaging modality best suited to assess morphology [21–23]. In patients with pulmonary atresia, there is frequently a need for exact hemodynamic and functional assessments. There currently is no substitute for invasive cardiac catheterization. Another limitation of MDCT is that it poses a risk of radiation exposure. However, when applied in clinically appropriate patients, CT has a potential benefit to the person that far outweighs the projected small stochastic risk of development of radiation-induced malignancy. Advances in CT technology over the past decade, such as tube-current modulation, iterative reconstruction etc., have resulted in marked reduction in effective radiation dose [24]. In addition, the judicious use of a preliminary MDCT study in some patients could obviate invasive catheterization, which has higher radiation exposure and complications [25], or could provide a global road map for the performance of cardiac catheterization that lead to substantial reduction of procedure time, radiation dose, and contrast dose [26].

### Study limitations

There are some limitations to the present study. Firstly, because small percentage of patients underwent complete repair in this study, the intracardiac abnormalities of most patients lack surgical confirmation. But the comparison among the three imaging modalities in the small subgroup of patients who underwent full repair provide sufficient data to reach a conclusion that MDCT is comparable to TTE in evaluation of intracardiac abnormality. Secondly, description and classification about the anatomy of PA may have no uniform criterion and the results of this study reflect the experience of our single center. However, this does not greatly affect our conclusions because visualization of the cardiovascular anatomy can be obtained impersonally at MDCT.

## Conclusion

In conclusion, MDCT may be superior to angiography for the global preoperative evaluation of the pulmonary vasculature and pulmonary blood supply. Moreover, MDCT could provide a global road map for the performance of cardiac catheterization, which leads to substantial reduction of procedure time, radiation dose, and contrast dose. MDCT can correctly delineate the overriding aorta and VSD, which may be comparable to TTE. MDCT is a reliable exploratory test for noninvasive assessment of global cardiovascular anatomy in patients with PA-VSD.
